# Inter-Professional Collaboration and Occupational Well-Being of Physicians Who Work in Adverse Working Conditions

**DOI:** 10.3390/healthcare9091210

**Published:** 2021-09-14

**Authors:** José Viruez-Soto, Roberto C. Delgado Bolton, Montserrat San-Martín, Luis Vivanco

**Affiliations:** 1 Department of Health Service, Government of La Paz, La Paz 12087, Bolivia; antonioviruez@hotmail.com; 2 Department of Diagnostic Imaging (Radiology) and Nuclear Medicine, University Hospital San Pedro and Centre for Biomedical Research of La Rioja (CIBIR), 26006 Logroño, Spain; rcdelgado@riojasalud.es; 3 Platform of Bioethics and Medical Education, Centre for Biomedical Research of La Rioja, 26006 Logroño, Spain; 4 National Centre of Documentation on Bioethics, Rioja Health Foundation, 26006 Logroño, Spain; 5 Department of Statistics and Operational Research, University of Granada, 52003 Melilla, Spain; momartin@ugr.es; 6 Faculty of Health Sciences, European Atlantic University, 39011 Santander, Spain

**Keywords:** inter-professional collaboration, occupational stress, patient care team, physicians, professionalism, workplace

## Abstract

Inter-professional collaboration, empathy and lifelong learning, components of medical professionalism, have been associated with occupational well-being in physicians. However, it is not clear whether this role persists in adverse working conditions. This study was performed to assess whether this is the case. These three abilities, and the self-perception of somatization, exhaustion and work alienation, were measured in a sample of 60 physicians working in a hospital declared to be in an institutional emergency. A multiple regression model explained 40% of the variability of exhaustion, with a large effect size (Cohen’s-*f*^2^ = 0.64), based on a linear relationship with teamwork (*p* = 0.01), and more dedication to academic (*p* < 0.001) and management activities (*p* < 0.003). Neither somatization nor alienation were predicted by empathy or lifelong learning abilities. Somatization, exhaustion, or alienation scores either explained empathy, inter-professional collaboration or lifelong learning scores. These findings indicate that, in adverse working environments, physicians with a greater sense of inter-professional collaboration or performing multi-task activities are more exposed to suffering exhaustion.

## 1. Introduction

Professionalism refers to the set of skills and values that, in the case of medicine, characterize the essence of humanism in professional work. Although technical knowledge and clinical skills are foundational to medical professionalism, values are central to the definition of professionalism and distinguish it from the concept of a mere clinical competence. In fact, there are three competencies recognized as specific elements of medical professionalism: inter-professional collaboration, empathy and lifelong learning abilities. Based on this premise, David Stern [1] defined medical professionalism as “a foundation of clinical competence, communication skills, and ethical and legal understanding, upon which the aspiration to professionalism and wise application of its principles are built: excellence, humanism, accountability, and altruism”. Recipients of the benefits of this professionalism on health and welfare are not only patients, but also practitioners and, ultimately, society as a whole. In the specific case of inter-professional collaborative abilities, research suggests that interdisciplinary healthcare teamwork reduces patient death rates, improves clinical care and patient satisfaction, and reduces costs [1].

The effort–reward imbalance (ERI) model is a theoretical model of a psychosocial work environment with adverse effects on health and well-being that focuses on a mismatch between high efforts spent and low rewards gained at work [2]. In the frame of the ERI model, medical professionalism is supported on a contract of social reciprocity, where the rewards can be provided not only in economic terms, but also in personal acknowledgement or professional recognition. In contrast, professionalism can be threatened by multiple factors such as work instability, the absence of perspectives of promotion, personal acknowledgements, or a lack of working improvement opportunities [3]. Furthermore, when harsh working environments are accompanied by scarce or inadequately distributed resources, keeping professionalism uncorrupted can become a daily odyssey [4]. Some authors have described these environments as “adverse working conditions” [5]. In healthcare settings, these environments usually combine three aspects: (a) lack of resources (insufficient, poorly managed, or misused); (b) lack of recognition (or support) from institutions, supervisors, or co-workers; and (c) prevalence of an organizational culture that places institutional benefits above patients. Considering that medicine is, by definition, an altruistic profession, the personal conflict that practitioners have to deal with becomes greater when it is accompanied with unsatisfied social demands [6]. It is possible that this situation is more stressful for those physicians who have stronger ethical values and professional abilities [7].

A notable disparity in the access to healthcare persists in Latin America, even after the recent efforts dedicated to trying to reduce it [8]. Such a difference is greater when comparing public and private institutions, or when treatments are paid from patients’ pockets [9]. Bolivia is one of the most visible cases of such inequalities [10]. Even though important efforts have been developed to restructure Bolivian public healthcare institutions [9], scarce available data indicate that the system as a whole maintains some of the lowest indicators in the region regarding healthcare quality and safety [11,12]. This situation acquired special resonance in 2016, when the National Ombudsman declared the main and the oldest public hospital of the capital city of La Paz, named “Hospital de Clínicas”, in institutional emergency [13]. This public announcement appeared after the publication of a dramatic report, carried by the representation of the Ombudsman of La Paz, warning of the terrible working conditions in this healthcare institution. Some of the deficiencies reported were that 50% of the equipment in the nineteen medical and surgical departments was obsolete; beds and mattresses were old; there was a lack of hygiene in rooms, restrooms and corridors; roofs leaked and walls were worn; a presence of mice; and a reduced number of healthcare workers to attend patients [14]. Based on these facts, the Ombudsman stated in a press conference that, “Given such a depressing and precarious situation, the hospital should not be considered a third level healthcare institution. It is obsolete and does not adapt to the needs of the population” [13]. This announcement arrived after several claims from workers and patients. The abovementioned situation acquires especial relevance taking into consideration that this institution was in charge of the health coverage of the poorest communities and was used as a teaching institution for physicians-in-training and medical students. Finally, in 2018, the government announced a USD two million investment to renovate obsolete public hospitals. However, the Bolivian healthcare professionals’ claims regarding the need to improve their training and working conditions are still pending [15]. In 2021 and in the middle of the COVID-19 pandemic, a more recent report signed by the new Ombudsman, Ms. Nadia Cruz Tarifa, highlighted that important renovations in this hospital are still a pending task [16].

Based on the aforementioned situation, an observational study was performed in “Hospital de Clínicas” of La Paz before the COVID-19 pandemic started. The following hypothesis was tested and confirmed: in adverse working conditions, physicians with higher indicators of professionalism (measured by empathy, inter-professional collaboration and lifelong learning abilities) are more exposed to suffer stress in their workplace.

## 2. Methods

Participants were 60 practitioners (75% of medical staff) of “Hospital de Clínicas” in La Paz, who participated anonymously and voluntarily. The study design, approved by an independent ethical committee (Ref. CEICLAR-PI-199), was authorized by the hospital’s administration. Only medical staff were included in the study. Medical students, physicians-in-training, other healthcare professionals, and physicians working for tertiary parties were excluded.

Self-perception of somatization, exhaustion and work alienation were used as main measures. The Scale of Collateral Effects (SCE) of the Questionnaire of General Labour Well-being was administered as a measuring instrument. The SCE is composed of three mini-scales: the scale of somatization (SS), with 5 items; the scale of exhaustion (SE), with 4 items; and the scale of work alienation (SA), with 4 items [17]. Each item of the abovementioned mini-scales starts with the following statement: “Currently, because of my work, I feel:” followed by one specific symptom. The SS measures the following symptoms: digestive disorder, back pain, insomnia, headache, and muscle tension. Symptoms included in the SE are work overload, emotional exhaustion, physical exhaustion, and mental saturation. Finally, the SA assesses bad mood, low personal fulfilment, depersonalized treatment, and frustration. The perception of each symptom is answered following a 7-point Likert-type scale, reflecting a daily frequency in the last week from 1 (never) to 7 (always).

In addition, inter-professional collaborative work between physicians and nurses (JSAPNC), the Jefferson Scales of Empathy (JSE), and physician’s lifelong learning (JeffSPLL), were used as multi-score measures of medical professionalism. The JSAPNC (15 items) is answered in a 4-point Likert scale from 1 (strongly disagree) to 4 (strongly agree) [18]. The JSE (20 items) is answered in a 7-point Likert scale from 1 (strongly disagree) to 7 (strongly agree) [19]. The JeffSPLL (14 items) is answered in a 4-point Likert scale from 1 (strongly disagree) to 4 (strongly agree) [20]. In the aforementioned scales, higher scores are associated with greater development of the elements measured. Finally, information on age, gender, specialty, professional experience (years), salary range, and weekly dedication (hours) to clinical, academic and management activities, were also collected in a socio-demographic form.

Following international recommendations [21], only psychometric measures with alpha coefficients equal to or higher than 0.70 were included in the analyses. Scales of somatization, exhaustion and work alienation were used as dependent variables. After normality was assessed, using Pearson’s chi-squared and Lilliefors–Kolmogorov–Smirnov tests, comparative analyses using non-parametric Mann–Whitney U tests were performed to determine differences on the abovementioned scales according to all variables collected. Effect size (*r*) was calculated following the formula described by Fritz, Morris and Richler [22] and Tomczak and Tomczak [23] for non-parametric tests. Following the recommendation of other authors [24], an *r*-value equal to 0.50 was considered as a large effect size with a crucial practical importance; equal to 0.30 was a medium effect size, with a moderate practical importance; and equal to 0.10 was a small effect size, with a negligible practical importance.

Regarding age and global scores on empathy, teamwork and lifelong learning, Spearman’s correlation analyses were performed in order to determine statistical associations between them and scores on somatization, exhaustion and work alienation. All variables showing statistical significance in comparative and correlation analyses were included in a multiple linear regression analysis as potential predictors of dependent variables. A regression model was accepted only if conditions of statistical inference (normality, zero mean, constant variance and uncorrelatedness of the residuals, in addition to linearity and absence of multi-collinearity) were met. In order to quantify the degree of practical significance of a model obtained, the effect size (Cohen’s-*f*^2^) was calculated: a value equal to or greater than 0.02 and smaller than 0.15 was interpreted as a small effect, equal to or greater than 0.15 and smaller than 0.35 was a medium effect, and equal to or greater than 0.35 was a large effect, following the interpretation criteria proposed by Cohen [25].

All analyses were performed using R statistical software, version 3.6.2, for Windows. The statistical analyses of the data also included multilevel [26], rstatix [27], lsr [28], and nortest [29] packages.

## 3. Results

The average age of participants in the sample was 45 (*SD* = 9) years old, with a range between 26 and 71 years old. By gender, 41 (68%) physicians were male. By specialties, 41 (68%) worked in medical and 19 (32%) in surgical departments. By salary, 18 (30%) had a monthly salary higher than USD 2000. By experience, 17 (30%) had more than 10 years of professional experience. By working dedication, 13 (22%) physicians indicated that they dedicated more than half of their 40 weekly working hours to clinics, 7 (12%) to academic activities (i.e., teaching and mentoring), and 8 (13%) to management.

All scales showed excellent psychometric properties (Table 1). On the one hand, correlation analyses showed a positive association between exhaustion and inter-professional collaboration (*ρ* = 0.29; *p* = 0.03). In contrast, neither exhaustion, alienation nor somatization showed a correlation with empathy, lifelong learning and age. A summary of these analyses is shown in Table 2. On the other hand, comparative analyses showed differences in somatization and exhaustion scores, but not in work alienation, by specialty, salary, or working dedication. No differences were observed in any of the three measures by gender or professional experience. A summary of these analyses is shown in Table 3.

**Figure 1:**
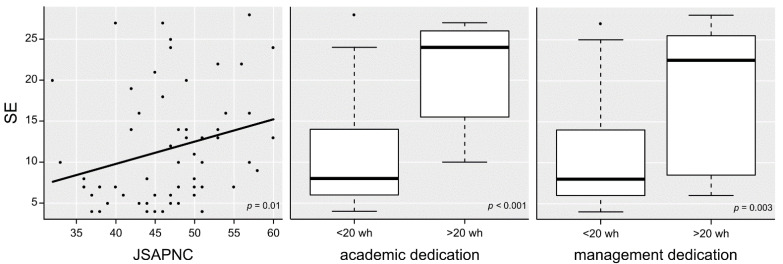
Variation in scores for the self-perception of exhaustion at workplace according to inter-professional collaboration, and weekly dedication to academic and management activities. SE, scale of exhaustion; JSAPNC, Jefferson Scale of Attitude towards Physician-Nurse Collaboration; wh, weekly hours.

Based on these preliminary findings, multivariate analyses were performed to determine interactions among the variables assessed. A one-way ANOVA confirmed a greater somatization only in physicians with more academic dedication (*p <* 0.001), with a large effect size (*η_p_*^2^ = 0.20). A three-way ANOVA confirmed something similar for exhaustion in physicians with more dedication to teaching (*p <* 0.001), with a large effect size (*η_p_*^2^ = 0.19); and in physicians working in medical departments (*p* = 0.02), and those with more management dedication (*p =* 0.01), both with a moderate effect size (*η_p_*^2^ *<* 0.14) (Table 4). Finally, two separate multiple linear regression analyses were performed for somatization and exhaustion, but only one fulfilled all necessary conditions for statistical inference. This model explained the 39% variance in exhaustion (R^2^-adjusted=0.36; *F*_(3,56)_ = 11.92; *p* < 0.001), with a large effect size (Cohen-*f*^2^ = 0.65). Inter-professional collaboration (*p* = 0.01), and more dedication to academic (*p <* 0.001) and management (*p =* 0.003) responsibilities appeared as predictors (Figure 1).

## 4. Discussion

In comparison with studies in Spain and other Latin American institutions, the three competences associated with medical professionalism measured in the Bolivian group were lower [18,19,20,30]. These findings could be caused by the negative influence that adverse working environments have on physicians’ professional performance and conduct, previously suggested in the literature [3,6], but also for some social and cultural characteristics that are dominant in Bolivian society. For example, in the case of empathy, findings observed are consistent with a progressive process of dehumanization reported in public Bolivian healthcare institutions [16,31], but also with some important gaps in physician–patient relationships associated with language barriers and cultural beliefs. These gaps are more evident in clinical encounters with patients from indigenous cultural backgrounds. The strong influence of a social stereotype supporting a vision of medicine above nursing can explain the low scores in inter-professional collaborative abilities in the entire sample. This vision is still predominant in the majority of Latin American institutions [6,32], where a paternalistic approach of medicine is also socially accepted. Regarding lifelong learning abilities, the poor development of these abilities correlates with findings reported in other studies, where healthcare professional with harsh working conditions and a lack of job support tend to suffer a lack of motivation towards excellence and professional accountability [33].

However, the most important finding of this study is the confirmation of the emotional and physical costs which those physicians with a greater sense of professionalism pay in adverse working environments. Unfortunately, the findings observed confirmed that having a higher development of professionalism increases the risk of suffering greater exhaustion. This finding is the opposite to those reported in other institutions, where in acceptable working conditions (even in those with some deficiencies), professionalism reduces work distress [6]. Moreover, collaborative physicians are able to encourage and motivate their co-workers in interdisciplinary teams. However, based on these findings, being more collaborative in adverse working conditions could play the opposite effect. It is reasonable to expect that more collaborative physicians are assuming more responsibilities than unmotivated ones. However, the reason why this effect is associated only to inter-professional collaboration but not to empathy or lifelong learning abilities can perhaps be explained by the retribution that these other abilities offer to the physicians which inter-professional collaborative abilities do not. For example, it is possible that more empathetic physicians handle the relationship with their patients better and receive moral support by the recognition and gratitude from the patients treated by them. This recognition could bring a meaning to their dedication that compensates, at least in part, the physical and emotional efforts in their daily work. In a similar sense, having a greater development of lifelong learning abilities usually derives from the improvements in clinical and technical competencies. It is plausible that physicians who are more skillful can receive better recognition from supervisors, co-workers, trainees and patients. In contrast, and in correlation with the ERI model [2], findings of this study indicate that more collaborative physicians with more dedication to academic or management activities do not perceive a benefit, neither moral nor professional, proportional to their efforts. Furthermore, the greater physical and emotional exhaustion observed in these physicians suggests the lack of an adequate balance between efforts dedicated and the reward received. These findings also coincide with what is proposed in the Job–Demand–Control–Support work model (JDCS model) regarding the negative effects derived from the lack of collaboration and social support from supervisors and colleagues in a work environment with high job demands [34]. According to the JDCS model, stress at work occurs when job demands exceed the ability to overcome those demands either through job control or job support, which, in normal conditions, act as moderators. When job stress, which is not directly measured by the JDCS model but is more of a latent variable thought to occur through the combination of job demands with job control and support, persists over a period of time, workers can experience the negative outcomes associated with stress, such as decreased physical health and exhaustion in their workplace, as these findings show.

## 5. Conclusions

In conclusion, in adverse working environments, for physicians with a high sense of responsibility or with a positive approach to inter-professional collaboration, work overload is not rewarded. For these physicians, the greater development of this ability poses the risk of taking them to assume not only their own responsibilities, but also those of their work team. The case reported in Bolivia poses a very serious and alarming problem, not only due to the risk for the health and welfare of physicians, but also due to the negative effects that not attending to this problem poses to other institutions associated with healthcare systems.

These findings suggest an urgent need to introduce important changes in the hospital where this study was performed, which not only affect facilities, but also the organizational culture of the institution. It is evident that there are important structural issues that should urgently be resolved. However, in the meantime, other aspects related to job support and working culture should be addressed in order to reduce the detrimental effect that this situation has in the health and wellbeing of physicians with greater sense of professionalism.

## Figures and Tables

**Table 1:** Descriptive analysis and reliability coefficients.

Statistics	JSE	JSAPNC	JeffSPLL	SS	SE	SA
Range possible	20–140	15–60	14–56	5–35	4–28	4–28
Range observed	81–134	32–60	31–56	5–33	4–28	4–24
Mean	101	47	47	13	12	9
Standard deviation (*SD*)	15	7	6	8	7	5
Quartile						
1st	88	42	44	8	6	5
2nd (Median)	94	47	48	10	10	7
3rd	114	51	51	18	16	13
Reliability	0.82	0.84	0.87	0.90	0.92	0.87

JSE, Jefferson Scale of Empathy; JSAPNC, Jefferson Scale of Attitudes toward Physician-Nurse Collaboration; JeffSPLL, Jefferson Scale of Physician Lifelong Learning; SS, scale of somatization; SE, scale of exhaustion; SA, scale of work alienation.

**Table 2:** Spearman’s correlation analysis among somatization, exhaustion, work alienation, empathy, inter-professional collaboration, lifelong learning and age.

	JSE	JSAPNC	JeffSPLL	SS	SE	SA	Age
JSE	1						
JSAPNC	+0.32 *	1					
JeffSPLL	+0.48 ***	+0.45 ***	1				
SS	+0.19	+0.24	+0.19	1			
SE	+0.13	+0.29 *	+0.21	+0.83 ***	1		
SA	+0.00	+0.21	+0.03	+0.54 ***	+0.65 ***	1	
Age	−0.19	+0.07	+0.11	−0.13	−0.19	−0.07	1

JSE, Jefferson Scale of Empathy; JSAPNC, Jefferson Scale of Attitudes toward Physician-Nurse Collaboration; JeffSPLL, Jefferson Scale of Physician Lifelong Learning; SS, scale of somatization; SE, scale of exhaustion; SA, scale of work alienation. * *p <* 0.05; *** *p <* 0.001.

**Table 3:** Summary result of Mann–Whitney U tests comparing scores on somatization and exhaustion by sex, specialty, salary, professional experience, and working dedication groups.

Study Groups	*n*	Somatization (SS)				Exhaustion (SE)			
		Mdn	M (*SD*)	*p*	*r*	Mdn	M (*SD*)	*p*	*r*
*Gender*									
Male group	41	12	13 (*7*)	0.85	--	8	11 (*7*)	0.50	--
Female group	19	9	13 (*8*)			10	12 (*7*)		
*Specialty*									
Medical branch	41	14	15 (*8*)	0.03	0.29	12	13 (*7*)	0.009	0.34
Surgical branch	19	9	10 (*4*)			7	8 (*4*)		
*Salary*									
Less than USD 2000	42	9.5	12 (*6*)	0.03	0.27	7	10 (*6*)	0.01	0.33
USD 2000 or more	18	16	17 (*9*)			13.5	16 (*8*)		
*Dedication to clinics*									
Less than 20 weekly hours	47	10	12 (*7*)	0.02	0.31	7	11 (*7*)	0.03	0.28
20 weekly hours or more	13	19	19 (*9*)			14	15 (*7*)		
*Dedication to academics*									
Less than 20 weekly hours	53	10	12 (*7*)	0.004	0.37	8	10 (*6*)	0.002	0.40
20 weekly hours or more	7	25	23 (*10*)			24	21 (*7*)		
*Dedication to management*									
Less than 20 weekly hours	52	10	13 (*7*)	0.10	--	8	11 (*6*)	0.02	0.30
20 weekly hours or more	8	17	18 (*10*)			22.5	18 (*9*)		
*Professional experience*									
Less than 10 years	43	10	14 (*8*)	0.62	0.06	10	11 (*6*)	0.92	--
10 years or more	17	10	13 (*8*)			9	12 (*8*)		

SS, scale of somatization; SE, scale of exhaustion; *n,* sample size; Mdn, median; M, mean; *SD,* standard deviation; *p*, probability; *r,* effect size.

**Table 4:** ANOVA of somatization and exhaustion measures by department, and academic and management weekly dedication as sources of variation.

Source of Variation	Somatization			
	*F*_(1,58)_	*η* ^2^	*η_p_* ^2^	*p*
Academic dedication (<20 weekly hours vs. >20)	14.52	0.20	0.20	<0.001
Source of variation	Exhaustion			
	*F*_(1,56)_	*η* ^2^	*η_p_* ^2^	*p*
Specialty (surgery vs. medicine)	5.58	0.06	0.09	0.02
Academic dedication (<20 weekly hours vs. >20)	13.28	0.15	0.19	<0.001
Management dedication (<20 weekly hours vs. >20)	6.73	0.07	0.11	0.01

*F*_(df)_, *F*-value (degrees of freedom); *η*^2^, eta-squared; *η_p_*^2^, partial eta-squared; *p*, probability.

## Data Availability

Data are available upon request.
